# Are Racial and Ethnic Disparities in Brachial Plexus Birth Injuries Explained by Known Risk Factors?

**DOI:** 10.21203/rs.3.rs-5363261/v1

**Published:** 2024-11-21

**Authors:** Mary Claire Manske, Machelle Wilson, Barton Wise, Herman Hedriana, Joy Melnikow, Daniel Tancredi

**Affiliations:** University of California Davis; University of California Davis; University of California Davis; University of California Davis; University of California Davis; University of California, Davis

## Abstract

**Objective:**

To investigate the association of maternal race/ethnicity with risk factors for brachial plexus birth injury (BPBI) and quantify the proportion of excess BPBI risk due to these factors.

**Study design:**

This retrospective cohort study of all livebirths occurring in California-licensed hospitals from 1996–2012 included 6,278,562 maternal-livebirth infant pairs with 7,762 BPBI diagnoses. Multiple logistic regression and causal mediation analyses were used to evaluate the relationship of race/ethnicity and BPBI risk factors.

**Results:**

Black and Hispanic birthing-individuals were at increased risk of obesity, diabetes, and limited prenatal care utilization but decreased risk of many BPBI risk factors (shoulder dystocia, macrosomia, prolonged second stage of labor, and vaginal delivery).

**Conclusions:**

Black and Hispanic birthing-individuals were at lower risk of many strongly associated risk factors for BPBI, and these factors mediate only a small proportion of their excess BPBI risk, underscoring the importance of identifying alternative risk factors, especially drivers of demographic disparities.

## Introduction

Brachial plexus birth injury (BPBI) is a traumatic injury to the nerve roots of the brachial plexus sustained by a newborn during labor and delivery believed to result from expulsive or traction forces on the brachial plexus nerve roots.^1^ It presents as upper extremity weakness or paralysis and occurs in approximately 1.5 per 1000 livebirths.^2–4^ Several risk factors for BPBI have been identified, including maternal factors (obesity, diabetes, gestational diabetes), infant factors (birthweight, macrosomia, post-date gestational age) and intrapartum factors (shoulder dystocia, instrumented delivery, vaginal delivery, prolonged labor, precipitous labor).^5^

Up to 30% of infants with BPBI have permanent nerve injuries,^6–10^ which result not only in persistent impairments in upper extremity motor and sensory function, but also the long term consequences of chronic denervation during skeletal immaturity, including impaired upper limb growth, joint contractures, and skeletal dysplasia.^11,12^ Many affected individuals experience lifelong disabilities in upper extremity and psychosocial function.^13^

Demographic disparities in BPBI risk exist, with Black and Hispanic birthing individuals at increased risk of delivering an affected infant.^3,4,14–18^ Compared to a population level incidence of 1.28 BPBI per 1000 livebirths, increased incidences of BPBI have been reported in Black and Hispanic infant deliveries, with 1.78 BPBI per 1000 livebirth among Black infants and 1.34 per 1000 per livebirth among Hispanic infants. Moreover, Black and Hispanic birthing individuals mothers have an 88% and 35% increased odds of delivering an affected infant, respectively, when adjusting for shoulder dystocia, macrosomia, delivery method and year of birth.^4^ However, the reasons for these demographic disparities are not well understood. In particular, the hypothesis that this disparity is due to increased risk of the known BPBI risk factors in these populations has not been confirmed. The purpose of this investigation was to evaluate if Black or Hispanic birthing individuals are at increased risk of the previously identified risk factors for BPBI, and to quantify how much of the excess risk of delivering an infant with BPBI is due to these factors.

## Methods

We obtained approval from the University of California Davis Institutional Review Board and the California Committee for the Protection of Human Subjects for this retrospective cohort study of all livebirth deliveries occurring in California-licensed hospitals from 1996–2012, the time period for which linked maternal-infant data is available. The study cohort was assembled from the state of California’s Department of Health Care Access and Information (HCAI) Linked Birth Files, which includes linked maternal and infant demographic and health information for 9 months prior to and 12 months post-delivery for all infants born in a California-licensed hospital from 1991–2012; after 2012, the state of California was unable to continue performing this linkage. This accounts for 98% of California births during this time period.^19^ The data are compiled by HCAI from the California Inpatient Discharge Dataset, Birth Certificate Dataset, and Vital Statistics Birth Cohort File. The dataset was created to facilitate research on pregnancy outcomes,^19–25^ and previous studies report its accuracy for maternal factors, intrapartum events, and obstetric complications.^26–28^

All maternal-infant pairs with an infant ICD-9 code indicating “livebirth” during an inpatient admission (V30, V31, V33, V34, V36, V37, V39) were included. Single and multiple gestation births were included, as were cesarean and vaginal deliveries. Unlinked mothers or infants, stillbirth infants, and mothers under 13 and older than 50 years were excluded. Maternal and infant demographic factors as reported on the discharge record and birth certificate: maternal age, infant sex, and race and ethnicity for both mother and infant. Missing maternal age at a given birth was imputed using the age at previous or subsequent births and the date of birth of the infant. Maternal age classified as young (≤ 19 years), reference (20–34 years), or advanced (≥ 35 years). Missing or unknown ethnicity was imputed as ‘Non-Hispanic’ for both the mother and infant. We restricted the cohort to deliveries occurring from 1996–2012 because the coding of race and ethnicity changed in 1995 to permit more detailed demographic characterization and was not consistently applied until 1996.

Our primary exposures of interest were maternal race (Black vs non-Black) and ethnicity (Hispanic vs non-Hispanic). The outcome variable for this study was infant diagnosis of BPBI, identified using ICD-9 codes (767.6 or 953.4). The mediators of interest included factors that have previously been associated with BPBI, all of which were available as variables in the dataset or using ICD-9 codes, including: maternal obesity, diabetes mellitus, gestational diabetes; Infant macrosomia (birthweight > 4000g), post-term delivery (> 40 weeks 6 days gestational age); shoulder dystocia, vaginal delivery, precipitous labor, and prolonged second stage of labor. We evaluated 2 additional potential risk factors: prenatal care utilization and insurance status. Prenatal care utilization was determined using the Adequacy of Prenatal Care Utilization (APNCU) Index, ^29^ which classifies prenatal care utilization as Inadequate, Intermediate, Adequate or Adequate Plus. This variable is calculated using gestational age at birth, number of prenatal care visits, and month prenatal care began, which were available in the HCAI data. We defined limited prenatal care utilization (Limited PCU) as Inadequate or Intermediate prenatal care utilization according to the APNCU Index, and adequate prenatal care utilization (Adequate PCU) as adequate or adequate plus prenatal care utilization. The multiple insurance types in our dataset were also categorized into two groups, Adequately Insured or Under Insured. The adequately insured category included Private insurance, Medi-Cal (state insurance program), and Medi-Care, while the underinsured category was defined as Self-pay, Worker’s Compensation, Indigent Programs or Other).

### Statistical analysis

Descriptive statistics were calculated for maternal and infant demographic factors and the known and suspected risk factors described above, including maternal obesity, diabetes, gestational diabetes, shoulder dystocia, macrosomia, vaginal delivery, post-term delivery, prolonged second stage of labor, precipitous labor, limited PCU, and underinsured. Multivariable logistic regression was used to determine the associations of BPBI with Black race and Hispanic ethnicity, controlling for maternal age, parity and year of infant birth; in the race model, we additionally controlled for ethnicity, and in the ethnicity model we additionally controlled for race. Multivariable logistic regression was also used to determine the associations of Black race and Hispanic ethnicity with known or suspected risk factors for BPBI, controlling for maternal age, parity, year of infant birth, and either ethnicity or race as appropriate. Factors for which Blacks or Hispanics were at increased risk were considered potential mediators of the BPBI-race/ethnicity relationship and included in the causal mediation analysis. Factors for which Black or Hispanic birthing individuals were at decreased risk were considered suppressors and not included in the causal mediation analysis.

Causal mediation analysis^30,31^ was used to quantify the proportion of excess BPBI risk in Black and Hispanic birthing individuals that is explained by each of the potential mediators ( factors for which Black and Hispanic birthing individuals were at increased risk). The mediators were coded so that the reference category was the “low-risk” category for each factor.

Causal mediation analysis provides estimates of the excess risk that is mediated or eliminated by each of the mediators. Percent excess BPBI risk mediated ( “percent mediated”) by a particular factor is the proportion of the excess BPBI risk attributable to that factor; in essence, how important a particular mediator is in explaining the effect of the exposure (race/ethnicity) on the outcome (BPBI). Percent excess BPBI risk eliminated (“percent eliminated”) is the proportion of excess risk that would be resolved if an intervention made the the effect of the mediator the same in each group and between-group differences in the effect of the risk factor were eliminated; in essence, if the effect of the factor was the same for Black and non-Black, or Hispanic and non-Hispanic individuals. ^32^

All statistical analysis was performed using SAS^®^ software version 9.4 for Windows^®^. (SAS Institute Inc, Cary, NC). Significance was established at p < 0.05. While this data set contains multiple births to the same individual, the intra-cluster correlation was low and hence ignored.^4^

## Results

Our cohort included 6,286,324 infants born to 4,104,825 individuals. 1,334,954 individuals contributed a single birth to the cohort and 2,769,871 contributed more than one birth. The mean number of infants per individual was 1.53 ± 0.83. BPBI was diagnosed in 7,762 infants (1.23 BPBI per 1000 livebirths). Maternal and infant demographic characteristics are included in Table 1; the association of these demographic characteristics with BPBI has been published previously.^4^ The distribution of known and suspected risk factors, stratified by race and ethnicity, is included in Table 2.

### Association of Race, Ethnicity, and risk factors with BPBI (Table 3)

After adjusting for maternal age, parity, and infant year of birth, Black and Hispanic birthing individuals had a 53.9% (aOR=1.539, 95% CI: 1.408, 1.682), and 19.5% (aOR=1.195, 95% CI: 1.140, 1.252) increased odds of delivering a BPBI infant, respectively. All the examined factors, except underinsured, were significantly associated with BPBI.

### Association of Race and Ethnicity with potential risk factors for BPBI (Table 4)

Compared to non-Black individuals, Black individuals had significantly increased odds of obesity and diabetes but decreased odds of many factors associated with BPBI, including gestational diabetes, shoulder dystocia, macrosomia, vaginal delivery, post-term delivery, prolonged second stage of labor, and precipitous labor. Black birthing individuals also had increased odds of limited prenatal care utilization and being underinsured.

Hispanic individuals had significantly increased odds of obesity, diabetes, gestational diabetes, and post-term gestational age, but significantly decreased odds of shoulder dystocia, macrosomia, vaginal delivery, prolonged second stage of labor and precipitous labor. Compared to non-Hispanic individuals, Hispanic mothers had increased odds of limited prenatal care utilization and being under insured.

### Mediation Analysis (Table 5)

In Black birthing-individuals, the percent excess BPBI risk that is mediated by obesity, diabetes, and limited prenatal care utilization is 8.77% (95% CI: 6.72, 10.83), 2.63% (95% CI: 1.92, 3.35), and 1.60% (95% CI: 0.89, 2.31), respectively. Among Hispanic birthing individuals, the percent excess BPBI risk mediated by obesity is 3.69% (95% CI: 2.61, 4.77), by diabetes is 5.18% (95% CI: 3.61, 6.75), by gestational diabetes is 7.69% (95% CI: 5.56, 9.81), by post-term delivery is 0.20% (95% CI: 0.09, 0.31), and by limited prenatal care utilization is 4.80% (95% CI: 2.51, 7.10).

## Discussion

Despite the well-documented racial and ethnic disparities in BPBI risk,^3,4,14–18^ the factors contributing to these differences are not well understood. A first step in addressing these inequalities is to evaluate if the established risk factors for BPBI account for the increased risk in these populations. If so, we could target these factors for prevention efforts in high-risk populations and reduce demographic disparities. In this study we found that Black and Hispanic individuals were at lower risk of many of the strongest risk factors for BPBI, including shoulder dystocia, macrosomia, vaginal delivery, prolonged second stage of labor and precipitous labor.

The fact that Black and Hispanic individuals are at lower risk of many of the most strongly associated factors associated with BPBI indicates that the observed increased risk of BPBI in Black and Hispanic individuals is often an underestimation of the adjusted disparity. When included in multivariable regression analyses, factors that are less likely in Black and Hispanic individuals exert a suppression effect on the race-BPBI or ethnicity-BPBI relationship. Far from explaining the causes of the disparities, the association of race/ethnicity with BPBI is actually more pronounced than previously understood. Consequently, interventions such as simulation training in the management of shoulder dystocia, which are effective at reducing the incidence of shoulder dystocia and BPBI overall, ^33,34^ are unlikely to resolve disparities by race or ethnicity in BPBI risk. This finding demonstrates the need to identify alternative risk factors for BPBI contributing to these disparities.

Another notable finding of this study is that Black and Hispanic individuals were at higher risk of obesity and diabetes or gestational diabetes, but not shoulder dystocia or macrosomia. Traditionally, obesity and gestational diabetes are thought to increase the risk of BPBI via their association with macrosomia and subsequent shoulder dystocia, especially in the setting of fetopelvic disproportion. However, our finding, combined with other studies demonstrating BPBI in the absence of shoulder dystocia, ^35–38^ suggests an alternative pathway for BPBI. Several previous investigations have proposed an in utero injury^35–38^ or fetal malposition^39^ as alternative mechanisms contributing to BPBI. Alternately, there may be a direct effect of hyperglycemia or other metabolic derangements on the brachial plexus’s susceptibility to injury.

Our third finding is that limited prenatal care utilization (PCU) is associated with a 14% increased odds of BPBI, and that Black and Hispanic birthing individuals were 26% and 40% more likely to have limited PCU, a finding consistent with other studies. ^40,41^ PCU is both identifiable prenatally and potentially modifiable, and therefore an ideal risk factor for BPBI, as this may be a more actionable risk factor than many intrapartum factors, such as shoulder dystocia. Although we did not have the ability to evaluate which components of prenatal care utilization are associated with decreased BPBI risk (e.g. diabetes management, fetal weight monitoring), this will be important to investigate in future studies.

Collectively, the above findings underscore the importance of identifying alternative risk factors for BPBI, especially those driving disparities. Although few studies have investigated the underlying causes of racial and ethnic disparities in BPBI risk, many studies have evaluated the factors contributing to demographic disparities in other adverse perinatal outcomes, including severe maternal morbidity, maternal mortality, maternal hemorrhage, neonatal morbidity and mortality, prematurity, low and very-low birth weight, intraventricular hemorrhage or necrotizing enterocolitis.^42,43,45–51^ Factors associated with racial and ethnic disparities in these conditions include: hospital characteristics (obstetric volume,^52–54^ NICU level and volume,^45^ urban vs rural location, teaching status,^46^ racial diversity of work force^55^),^43,45,46,48,49,49,51–54,56–58^ provider factors (provider delivery volume,^59^ racial concordance^60^), social determinants of health (insurance status, maternal education),^47,61^ and structural racism.^62^ Interestingly, there is a tendency for higher risk racial and ethnic groups to deliver at poorer performing hospitals with higher risk-adjusted morbidity and mortality rates, conferring additional systems-level disadvantage, in addition to individual level risk factors.^46,48–50^ In addition to pregnancy and obstetric related factors discussed earlier, social determinants of health, provider level factors, and systems level factors such as hospital characteristics and healthcare workforce factors should be investigated for their association with BPBI, and may be viable targets for prevention at the individual, systems, or healthcare policy level. Because observed racial and ethnic disparities are likely reflective of historic social, cultural, and economic inequities, rather than differences in physiology and biology, it is possible that potential solutions for observed inequities lie in the social, culture, economic, and healthcare system domains, rather than exclusively in the biologic domain at the individual level.

Limitations of this study include the retrospective study design and limitations intrinsic to administrative datasets, including that the source files were created for medical billing and resource allocation. Some variables potentially relevant to BPBI risk (e.g maternal weight gain, body mass index, medical comorbidities and estimated fetal weight) were not available. Other clinically relevant prenatal information, such as fetal ultrasound measurements or other biomarkers (e.g. hemoglobin A1c), were also not present in the dataset and, therefore, unable to be analyzed. Our cohort included only infants diagnosed with BPBI at birth; infants with mild BPBI injuries not diagnosed at birth may not have been included. However, we believe the accuracy for this diagnosis in the dataset is likely very high, given that BPBI is most commonly diagnosed at birth, is not readily mistaken for another diagnoses, does not require confirmatory testing, and has unique ICD-9 codes. Another limitation is that our data set ends in 2012 because the state of California no longer performed the linkages between birthing individuals and infants needed for these analyses; however, analyses of more contemporary datasets, albeit with infant only data, indicate similar longitudinal trends in BPBI incidence beyond 2012 as those observed in this dataset.^3,4,63^

Strengths of this study include the large size of the dataset, the diversity of maternal-infant pairs, its continuous coverage over a 17-year period, and demonstrated accuracy for obstetric complications and birth diagnoses.^26–28^ The use of linked maternal-infant data improves upon studies using infant-only or maternal-only data which often do not provide sufficient information to evaluate the effect of maternal history or factors on infant conditions.^64,65^ Additionally, this dataset compiles data from several sources, which allows cross-checking accuracy among overlapping variables and broader coverage from non-overlapping variables.

In this study we found that despite being at increased risk of delivering an infant with BPBI, Black and Hispanic birthing individuals are at decreased risk of many of the strongest and most common risk factors for BPBI. Factors for which they are at increased risk account for a small proportion of the excess risk. To address the observed disparities, it is imperative to identify the factors driving these inequities and develop prevention strategies aimed at these factors. Given that race and ethnicity are constructs influenced by historic social, culture and economic factors, potential drivers of the observed disparities in BPBI include social, cultural, economic, and healthcare systems factors at both the individual and societal level that have been implicated in racial and ethnic inequalities observed in other adverse perinatal outcomes.

## Figures and Tables

**Table 1:** Demographic Characteristics of Study Cohort

Maternal Characteristics N=6,286,324		
		Mean (SD)
**Age** (years)	n=6,286,324	28.3 (6.2)
		**No. (%)**
**Age Category**	n=6,286,324	
< 19 years		506,964 (8.06%)
20–34 years		4,905,359 (78.03%)
> 35 year		874,001 (19.3%)
**Race**	n=6,286,324	
Asian		718,588(11.43%)
Black		325,949(5.19%)
Native American		28,509(0.45%)
White		4,095,319(65.15%)
None of the above		1,117,959(17.78%)
**Ethnicity**	n=6,239,875	
Hispanic		2,723,422 (44.06%)
Non-Hispanic		3,516,453 (55.94%)
**Parity**		
n=6,286,324		
Primiparas		3,516,453 (55.94%)
Multiparas		2,769,871 (44.06%)
**Number of previous deliveries** n=6,286,324		
0		3,516,453 (55.94%)
1		1,757,348 (27.96%)
2		696,987 (11.09%)
3		217,198 (3.46%)
4		64,713 (1.03%)
5		33,625 (0.53%)
**Infant Characteristics**		
Total		**No. (%)**
**Sex** n=6,286,324		
Male		3,181,664 (50.61%)
Female		3,104,612 (49.39%)
**Race**	n=6,286,321	
Asian		688,770 (10.96%)
Black		319,012 (5.07%)
Native American		27,506 (0.44%)
White		4,126,162 (65.64%)
None of the above		1,124,871 (17.89%)
**Ethnicity**	n=6,286,324	
Hispanic		2,667,685 (42.44%)
Non-Hispanic		3,618,639 (57.56%)

**Table 2:** Distribution of Risk Factors Strati ed by Race and Ethnicity

	Black N (%)	Non-Black N (%)	p-value	Hispanic N (%)	Non-Hispanic N (%)	p-value
Obesity	12,037 (3.69)	102,474 (1.72)	<.001	51,636 (1.9)	62,875 (1.76)	<.001
Diabetes	2,307 (0.71)	34,348 (0.58)	<.001	18,467 (0.68)	18,188 (0.51)	<.001
Gestational Diabetes	12,203 (3.74)	318,436 (5.34)	<.001	150,995 (5.54)	179,644 (5.04)	<.001
Shoulder Dystocia	763 (0.23)	15,012 (0.25)	.048	5,779 (0.21)	9,996 (0.28)	<.001
Macrosomia	17,116 (5.25)	502,614 (8.43)	<.001	224,241 (8.23)	295,489 (8.29)	.007
Vaginal Delivery	230,900 (70.84)	4,385,754 (73.58)	<.001	2,019,939 (74.17)	2,596,715 (72.88)	<.001
Post-term	13,012 (3.99)	254,125 (4.26)	<.001	121,399 (4.46)	145,738 (4.09)	<.001
Prolonged labor	1,088 (0.33)	53,400 (0.90)	<.001	13,875 (0.51)	40,613 (1.14)	<.001
Precipitous labor	10,650 (3.27)	189,588 (3.18)	.006	81,167 (2.98)	11,9071 (3.34)	<.001
Limited Prenatal Care	82,616 (25.35)	1,334,976 (22.40)	<.001	712,080 (26.15)	705,512 (19.80)	<.001
Under Insured	19,961 (6.15)	276,622 (4.66)	<.001	130,124 (4.80)	166,459 (4.70)	<.001

**Table 3:** Unadjusted and Adjusted Odds of BPBI for each Risk Factor

	OR (95% CI)	p value	AOR (95%CI)	p value
Obesity	2.37 (2.13, 2.65)	<.001	2.93 (2.62, 3.28)	<.0001
Diabetes	4.34 (3.76, 5.01)	<.001	4.47 (3.87, 5.16)	<.0001
Gestational Diabetes	2.20 (2.05, 2.36)	<.001	2.40 (2.23, 2.58)	<.0001
Shoulder Dystocia	70.72 (66.22, 75.52)	<.001	64.20 (60.03, 68.66)	<.0001
Macrosomia	7.75 (7.41, 8.12)	<.001	7.52 (7.19, 7.88)	<.0001
Vaginal Delivery	7.31 (6.58, 8.12)	<.001	7.21 (6.49, 8.01)	<.0001
Post-term	1.40 (1.28, 1.54)	<.001	1.30 (1.19, 1.43)	<.0001
Prolonged labor	3.38 (2.95, 3.86)	<.001	3.32 (2.90, 3.80)	<.0001
Limited Prenatal Care	1.18 (1.12, 1.24)	<.001	1.14 (1.08, 1.20)	<.0001
Under Insured	0.85 (0.76, 0.95)	.005	0.90 (0.80, 1.00)	0.0596
Year of Birth	0.944 (0.939, 0.949)	<.001	1.059 (1.053,1.064)	<.0001

AOR=Adjusted Odds Ratios were each estimated in a unique model, controlling for maternal age, race, ethnicity, parity and year of infant birth.

**Table 4:** Adjusted Odds of each Risk Factor by Maternal Race and Ethnicity

Risk Factor	Black Race AOR (95%CI)	p	Hispanic Ethnicity AOR (95%CI)	p
Obesity	2.43 (2.38,2.48)	<0.0001	1.29 (1.27, 1.31)	<0.0001
Diabetes	1.57 (1.50,1.64)	<0.0001	1.63 (1.59, 1.66)	0.0001
Gestational Diabetes	0.84 (0.82,0.85)	<0.0001	1.27 (1.26, 1.28)	0.0001
Post-term Delivery	0.95 (0.93,0.96)	<0.0001	1.03 (1.02, 1.04)	<0.0001
Vaginal Delivery	0.79 (0.78,0.79)	<0.0001	0.969 (0.966, 0.973)	<0.0001
Shoulder Dystocia	0.76 (0.71,0.82)	<0.0001	0.75 (0.73, 0.78)	<0.0001
Macrosomia	0.60 (0.59,0.61)	<0.0001	0.99 (0.98, 1.00)	0.0018
Prolonged 2^nd^ stage Labor	0.33 (0.31,0.35)	<0.0001	0.38 (0.37, 0.39)	<0.0001
Precipitous Labor	0.88 (0.86,0.90)	<0.0001	0.92 (0.91,0.93)	<0.0001
Limited Prenatal Care Utilization	1.26 (1.25,1.27)	<0.0001	1.406 (1.400, 1.411)	<0.0001
Under Insured	1.29 (1.28,1.32)	<0.0001	1.04 (1.03, 1.05)	<0.0001

AOR=Adjusted Odds Ratios were each estimated in a unique model, controlling for maternal age, parity and year of infant birth. In the Black Race model, we additionally controlled for ethnicity. In the Hispanic Ethnicity model, we additionally controlled for race.

**Table 5:** Percent Excess BPBI Risk Mediated and Eliminated for Mediator stratified by Race and Ethnicity

Black Race				
Mediator	Percent Mediated (95%CI)	p	Percent Eliminated (95%CI)	p
Obesity	8.77 (6.72, 10.83)	<0.0001	10.85 (8.58, 13.11)	<0.0001
Diabetes	2.63 (1.92, 3.35)	<0.0001	4.24 (3.32, 5.16)	<0.0001
Limited Prenatal Care Utilization	1.60 (0.89, 2.31)	<0.0001	4.47 (2.62, 6.31)	<0.0001
Hispanic Ethnicity				
Mediator	Percent Mediated (95%CI)	p	Percent Eliminated (95%CI)	p
Obesity	3.69 (2.61, 4.77)	<0.0001	5.75 (4.47, 7.03)	<0.0001
Diabetes	5.18 (3.61, 6.75)	<0.0001	6.48 (4.78, 8.18)	<0.0001
Gestational Diabetes	7.69 (5.56, 9.81)	<0.0001	12.19 (9.86, 14.52)	<0.0001
Post-term Delivery	0.20 (0.09, 0.31)	0.0003	0.19 (0.83, 1.95	<0.0001
Limited Prenatal Care Utilization	4.80 (2.51, 7.10)	<0.0001	7.30 (4.12, 10.49)	<0.0001
